# A 360-degree imagery-multisensor system for visualizing environmental parameters in architecture and urban spaces

**DOI:** 10.1016/j.ohx.2025.e00643

**Published:** 2025-03-25

**Authors:** Mojtaba Parsaee, André Potvin, Jean-François Lalonde, Marc Hébert, Claude M.H. Demers

**Affiliations:** a Department of Built Environment, Indiana State University, Terre Haute, United States; b School of Architecture, Laval University, Quebec, Canada; c Department of Electrical and Computer Engineering, Laval University, Quebec, Canada; d Faculty of Medicine, Laval University, Quebec, Canada

**Keywords:** Built Environment, Environmental Design, Data Capture, Visualization, Panoramic Image

## Abstract

This research has designed a 360-degree imagery-multisensor system aiming to capture and visualize environmental parameters in architecture and urban spaces. Unlike existing tools, this system enables simultaneous recording of both imagery and non-imagery environmental data, including lighting, thermal, air quality, sound, and physical space parameters, within a 360-degree field of view. Lighting conditions are captured using panoramic high dynamic range imagery, complemented by a 360-degree array of sensors measuring illuminance levels and spectral power distribution. Thermal and air quality conditions are recorded using 360-degree thermal imagery, combined with hygrometers and air particle meters. Sound levels are also monitored across the full 360-degree field. The system is built using 3D printing technologies and Raspberry Pi computers, equipped with various sensor modules. Custom Python scripts enable real-time data capture, processing, and visualization. This cost-effective, easy-to-manufacture, programmable, and customizable innovation is aimed at students and educators in design and architecture, as well as building engineers. Furthermore, integrating imagery and sensor data supports the development of immersive virtual and augmented reality applications, offering new opportunities for education and the exploration of effective design solutions.

## Graphical abstract

**Figure:**
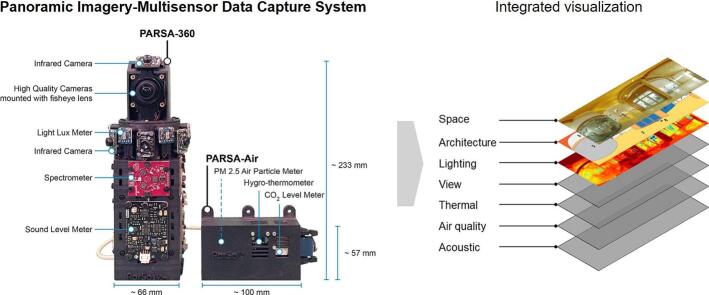

Specifications table

**Table:** 

Hardware name	**Panoramic And Real-time Sensor Array (PARSA-360 + Air)**
Subject area	• Educational tools • Architecture • Building Engineering • Urban design
Hardware type	• Imaging tools • Measuring physical properties in buildings and urban spaces • Panoramic visualization of environmental qualities, i.e., lighting, thermal, air quality, and acoustic, in architecture and urban spaces • Field measurements and sensors
Open-source license	This work is licensed under the Creative Commons Attribution-Share Alike 4.0 International License (CC BY-SA 4.0). To view a copy of this license, visit https://creativecommons.org/licenses/by-sa/4.0/
Cost of hardware	CAN$1,666.96 (subject to change by the time and location)
Source file repository	This device is made of two components shared via the following OSF Repository.https://doi.org/10.17605/OSF.IO/QB7PH

## 1 Hardware in context

**Fig. 1:**
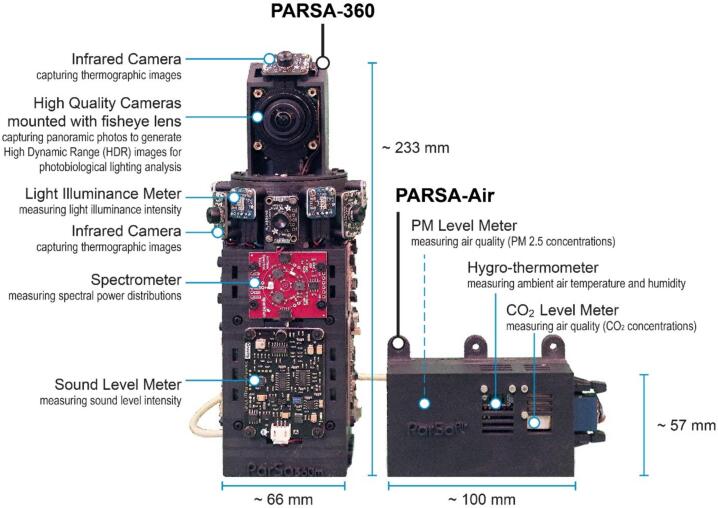
A prototype of the developed ‘Panoramic And Real-time Sensor Array’ (PARSA 360 + Air) system.

This paper presents the development of a 360-degree imagery-multisensor capturing system named **‘Panoramic And Real-time Sensor Array’** (**PARSA-360 + Air)** as shown in Fig. 1. This system enables the simultaneous capturing and visualization of both imagery and non-imagery environmental parameters, including lighting, thermal, air quality, acoustic, and spatial attributes, within a 360-degree field of view. PARSA-360 + Air is specifically designed for applications in architecture, building science, and urban design.

### 1.1 Comparison with existing literature and devices

Capturing and visualizing environmental qualities from a human-level perspective is critical for advancing the development of healthy, energy-efficient, and nature-friendly buildings, communities, and cities. Previous studies, such as Potvin [1], [2], Demers and Potvin [3], Boiné, et al. [4], have highlighted the importance of measuring and visualizing indoor and outdoor environmental parameters to promote sustainability and occupant well-being. Numerous researchers, including Karami, et al. [5], Ali, et al. [6], Jin, et al. [7], Amirazar, et al. [8], Botero-Valencia, et al. [9], Rhudy, et al. [10], have developed open-source tools and devices to measure key indoor environmental quality parameters. These parameters include lighting, hygro-thermal conditions (i.e., air and surface temperature, relative humidity), air quality (i.e., carbon dioxide (CO_2_) and particulate matter (PM2.5) concentrations), and sound levels. Prior research, such as Potvin [1], [2], Pineda-Tobón, et al. [11], Botero-Valencia, et al. [12], have also focused on developing open-source devices to capture outdoor environmental parameters such as lighting, thermal conditions, air quality, and acoustics. Smartwatches have been utilized to collect physiological and environmental data in urban environments, providing valuable insights into the interaction between people and city spaces [13]. These contributions underscore the significance of integrating environmental data visualization for various critical purposes as discussed in the following sub-sections.

The PARSA-360 + Air system distinguishes itself from existing open-source tools and devices for measuring indoor and outdoor environmental quality, such as [1], [2], [5], [6], [7], [8], [9], [10], [11], [12], [13], by offering integrated, simultaneous capture of imagery and non-imagery environmental parameters (lighting, thermal conditions, air quality, acoustics, and spatial attributes) within a full 360-degree field of view. Unlike devices that focus on individual parameters or lack spatial contextualization [1], [2], [5], [6], [7], [8], [9], [10], [11], [12], [13], PARSA-360 + Air integrates panoramic imagery, enabling users to correlate environmental measurements with specific locations and features within a space. Furthermore, its low-cost design, ease of manufacturing using 3D printing, and customizable Python-based programming make it more accessible and adaptable than some commercial systems, providing a valuable tool for researchers, educators, and designers interested in holistic environmental analysis. This integrated approach facilitates the creation of immersive virtual and augmented reality experiences for studying human responses to architectural design, assessing energy efficiency, and promoting occupant well-being.

### 1.2 Integrated measurement and capture of environmental parameters

The ability to capture and visualize both imagery and non-imagery environmental parameters from an observer’s perspective is critical for advancing the understanding of the complex relationships between architecture, urban design, health, well-being, and energy efficiency [1], [2], [3]. By integrating environmental data into a single visualization framework, designers, researchers, and decision-makers can more effectively analyze how various parameters, such as lighting, thermal conditions, air quality, acoustics, and spatial attributes, interact with each other and with human perception [1], [2], [3], [7]. Integrated visualization offers a holistic approach to understanding environmental performance at human scales, enabling comprehensive insights that go beyond isolated measurements [14], [15]. This approach facilitates the identification of data patterns and correlations to create healthier, more sustainable, and energy-efficient environments, such as how lighting quality impacts energy consumption or how air quality affects thermal comfort [14], [15]. Integrated environmental visualization also serves as a powerful educational tool [6], [14], [15]. By presenting environmental data in a visually engaging and intuitive manner, it helps communicate complex concepts to diverse audiences, including students, educators, and community stakeholders [1], [2], [3], [6]. This approach fosters greater awareness of the importance of environmental qualities and encourages collaborative efforts toward designing spaces that promote human health and sustainability [1], [2], [3], [6]. Recent studies have emphasized the critical need for comprehensive data collection, encompassing both imagery and non-imagery environmental information, to effectively analyze urban design and human perceptions [16], [17], [18].

### 1.3 Immersive visualization of buit environment parameters

Utilizing panoramic imagery and sensing technology to create immersive virtual environments is instrumental in engaging various stakeholders, including students and educators, in exploring and understanding the effects of environmental and design factors on health, well-being, and energy efficiency [19], [20]. 360-degree imagery is a foundational component of immersive experiences in virtual, augmented, and mixed realities [20]. These technologies allow users to virtually enter existing spaces without physically being present. Augmented and mixed realities further enrich this experience by overlaying or altering elements within these spaces [21], [22]. Combining 360-degree photography with digital simulations of environmental qualities has gained increasing attention in architectural, engineering, and urban design applications [21], [22]. Recent studies highlight the potential of these technologies in investigating physiological, psychological, and emotional responses to architectural design features, including lighting, thermal, acoustic, and spatial parameters [20], [23], [24].

### 1.4 Measurement and visualization of lighting characteristics

Designing lighting conditions to optimize occupants’ health and well-being requires detailed characterization of light distribution and spectral properties at the human eye level [25], [26]. High Dynamic Range (HDR) imaging, combined with illuminance measurements and spectrophotometry, provides an advanced method for analyzing lighting qualities and architectural configurations [27], [28]. HDR images are created by capturing multiple Low Dynamic Range (LDR) images, acquired using common commercial cameras and lenses, with varying exposure values (EV), typically ranging from −3 EV (very dark) to + 3 EV (very bright) [26], [29], [30]. These bracketed images are then fused together to generate a single HDR image that captures a wider range of luminance values than a standard LDR image can achieve on its own [26], [29], [30]. This technique allows for the simultaneous capture of details in both the darkest and brightest areas of a scene, resulting in an image that more closely resembles what the human eye perceives [27], [28]. HDR images enable the pixel-based evaluation of photobiological lighting parameters such as photopic, melanopic, and correlated color temperature (CCT) metrics [27], [28]. Illuminance meters are used to measure the intensity of light on surfaces or as perceived by the human eye, serving as a fundamental metric for designing natural and artificial lighting [25], [31], [32]. Spectrophotometry adds further insight by analyzing spectral power distributions and surface reflectance in relation to the photopic and melanopic sensitivities of human vision [25], [31], [32].

### 1.5 Measurement and visualization of thermal and air quality parameters

Thermal and air quality parameters are vital in ensuring human health, comfort, and productivity in indoor and outdoor environments [32], [33], [34], [35], [36]. These parameters are key to designing energy-efficient buildings, maintaining optimal living conditions, and minimizing health risks associated with poor air quality or inadequate thermal regulation [32], [33], [34]. Thermal parameters are assessed using a combination of hygro-thermometer measurements and infrared (IR) thermographic imaging [19], [32]. Hygro-thermometers measure ambient air temperature and humidity, providing fundamental data for evaluating thermal comfort and conditions in space. These measurements are essential for designing and maintaining environments with optimal thermal performance [14], [15]. Complementing hygro-thermometer data, thermographic imaging visualizes surface temperatures by detecting infrared radiation [19]. This method is widely used to identify heat losses, thermal bridges, and air leakage in buildings, such as through poorly insulated walls or windows [37], [38]. Thermographic imaging also aids in monitoring occupant thermal comfort and visualizing the distribution of body temperatures in various settings [37], [38].

Air quality is primarily evaluated by monitoring CO_2_ and PM2.5 levels [39], [40]. High CO_2_ and PM2.5 concentrations often indicate inadequate ventilation which can lead to reduced cognitive performance, lower discomfort, and health risks, including respiratory and cardiovascular issues [35], [36], [39], [40]. Visualization of air quality data involves mapping sensor measurements within spatial contexts, enabling the identification of problem areas and the development of targeted interventions [7], [41]. Combining hygro-thermometer measurements, thermographic images, and air quality data provides a comprehensive view of thermal conditions, enabling designers and engineers to develop targeted solutions for improving energy efficiency and occupant comfort.

### 1.6 Measuring and visualizing acoustic parameters

Acoustic parameters are critical for assessing the sound condition in different spaces, as they directly impact human comfort, health, and productivity [42], [43]. Excessive noise levels, usually above 90 dB (dB), can cause discomfort, stress, hearing loss, reduced productivity, and even long-term health issues, highlighting the importance of measurement and visualization to create optimal soundscapes [42], [44]. Sound level measurements can help evaluate noise intensity relative to human auditory thresholds [44]. Visualization of acoustic data includes representing sound intensity and distribution around an observer or within a space [42], [44]. These visualizations are invaluable for architects and engineers in designing interventions such as soundproofing, acoustic panels, or optimal spatial layouts to mitigate noise and enhance acoustic quality.

## 2 Hardware description

PARSA-360 + Air is an innovative system for capturing and visualizing environmental data, including imagery and non-imagery parameters such as lighting, thermal conditions, air quality, acoustics, and physical space attributes, within a 360-degree field of view. The system is designed to be low-cost, portable, autonomous, and customizable, ensuring easy replication by architects, designers, students, educators, and individuals without technical expertise in electronics or computer engineering. The system was developed by an architectural team using standard equipment found in architectural schools. Base boxes and lids are 3D printed with machines commonly available in architecture and design schools. The electronic assembly is simplified, with all steps fully documented and illustrated. The system uses Raspberry Pi microcomputers integrated with sensor modules and cameras, controlled by customizable Python programs designed for non-experts. The Raspberry Pi’s graphical user interface allows users to modify programs, operate modules, and process data, making the system particularly user-friendly for students, educators, architects, and building engineers. Users have complete control over the Raspberry Pi, sensors, and cameras, which can operate independently or simultaneously in the background. Data can be seamlessly captured, processed, and transferred to other desktop or external platforms for post-processing and deep-learning workflows. Replaceable sensors allow customization to meet diverse user needs. The 3D models for base boxes and lids can be adjusted for compatibility with different printing machines and materials.

Overall, PARSA-360 + Air offers the following applications and possibilities compared to previously developed open-source systems and hardware such as [1], [2], [5], [6], [7], [8], [9], [10], [11], [12].

• Enable real-time, immersive visualization of environmental features, using panoramic imagery and non-imagery data, including lighting, thermal, air quality, acoustic, and physical space attributes, to create highly engaging augmented and mixed reality experiences.
• Serve as a personalized monitoring system, informing individuals about the quality of the physical environments they interact with.
• Promote a deeper understanding and presentation of architectural and urban spaces, highlighting their complexities and how they impact individual experiences.
• Offer the potential to connect and synchronize with the Internet of Things (IoT), enabling the system to control and improve indoor environmental qualities based on occupant experiences and exposures.
• Provide rich datasets that can be integrated with point clouds produced by photogrammetry or LiDAR (light detection and ranging) scanners, enabling deep learning processes to reconstruct and analyze
• Serve as a powerful educational tool for students, educators, and researchers, enabling hands-on learning in environmental monitoring and data visualization for the architectural and urban design process.

## 3 Design files summary

Our hardware consists of two main units: (1) PARSA-360 and (2) PARSA-Air (see Fig. 1). Each unit is made up of several components, as outlined in Table 1. The files required for 3D printing and operating each unit are available through the open-access database link provided in Table 1.

**Table 1:** The list of files required to build and run PARSA 360 + Air.

**Design file name**	**File type**	**Open-source license**	**Location of the file**
PARSA360_BaseBox	STL, STEP, OBJ and 3DM	CC BY-SA 4.0	https://doi.org/10.17605/OSF.IO/QB7PH[45]
PARSA360_CamBase	STL, STEP, OBJ and 3DM	CC BY-SA 4.0	https://doi.org/10.17605/OSF.IO/QB7PH[45]
PARSA360_Cover	STL, STEP, OBJ and 3DM	CC BY-SA 4.0	https://doi.org/10.17605/OSF.IO/QB7PH[45]
PARSA360_Lid	STL, STEP, OBJ and 3DM	CC BY-SA 4.0	https://doi.org/10.17605/OSF.IO/QB7PH[45]
PARSAAir_BaseBox	STL, STEP, OBJ and 3DM	CC BY-SA 4.0	https://doi.org/10.17605/OSF.IO/QB7PH[45]
PARSAAir_Lid	STL, STEP, OBJ and 3DM	CC BY-SA 4.0	https://doi.org/10.17605/OSF.IO/QB7PH[45]
Installation	SH	MIT License	https://github.com/parsaeemojtaba/ParSa360.git[46]
Libs	Directory	MIT License	https://github.com/parsaeemojtaba/ParSa360.git[46]
PARSA360Air_Plotting	PY	MIT License	https://github.com/parsaeemojtaba/ParSa360-Air-Plotting.git[47]
Plot_libs	Directory	MIT License	https://github.com/parsaeemojtaba/ParSa360-Air-Plotting.git[47]

The files and directories are briefly described as follows:

• **PARSA360_BaseBox**: Contains the 3D model of the base box (frame) designed to mount sensors and Raspberry Pis.
• **PARSA360_CamBase**: Contains the 3D model of the base frame for mounting fisheye cameras.
• **PARSA360_Cover**: Contains the 3D model of the cover designed for Raspberry Pis.
• **PARSA360_Lid**: Contains the 3D model of the lid used to enclose the PARSA360 base box.
• **PARSAAir_BaseBox**: Contains the 3D model of the base box (frame) designed to mount the hygro-thermometer (temperature and humidity sensors) and air quality meters.
• **PARSAAir_Lid**: Contains the 3D model of the lid used to enclose the PARSAAir_BaseBox.
• **Installation**: Includes command lines for installing the libraries and dependencies required to run PARSA360 + Air and the associated sensors.
• **Libs**: A directory that contains the files, programs, and libraries needed to operate the sensors and capture data.
• **PARSA360Air_Plotting**: A Python program for plotting the captured data and images from sensors and cameras.
• **Plot_libs**: A directory containing the files, programs, and libraries necessary for running PARSA360Air_Plotting.

## 4 Bill of materials summary

**Table 2:** Bill of materials used to build PARSA 360 + Air.

Designator	Component	Number	Cost per unit – Currency: Canadian Dollar	Total cost − currency	Source of materials	Material type
3D printed parts						
PARSA360_BaseBox	Base box of PARSA360	1 (179 ml)	CAN$109.00 per 1 L	CAN$30.00	Link-1	Resin
PARSA360_CamBase	Camera base of PARSA360	1 (40 ml)	CAN$109.00 per 1 L	CAN$10.00	Link-1	Resin
PARSA360_Cover	A cover for the second Raspberry Pi	1 (20 ml)	CAN$109.00 per 1 L	CAN$5.00	Link-1	Resin
PARSA360_Lid	Lid of PARSA360	1 (31 ml)	CAN$109.00 per 1 L	CAN$10.00	Link-1	Resin
PARSAAir_BaseBox	Base box of PARSAAir	1 (53 ml)	CAN$109.00 per 1 L	CAN$15.00	Link-1	Resin
PARSAAir_Lid	Lid of PARSAAir	1 (50 ml)	CAN$109.00 per 1 L	CAN$10.00	Link-1	Resin
Electronic parts						
RaspiPi	Raspberry Pi 4, 4 GB RAM- Basic Kit Note that any lower version of Raspberry Pi can also be used.	2	CAN$55.00	CAN$110.00	Link-2	Semiconductor
HeatSink	Set of 3 aluminum heat sinks for Raspberry Pi’s	2	CAN$4.95	CAN$9.90	Link-3	Aluminum
MicroSD	Micro SD card (above 64 GB)	2	CAN$13.99	CAN$27.98	Link-4	Semiconductor
PowerSupply	USB-C power supply for Raspberry Pi	1	CAN$9.95	CAN$9.95	Link-5	Semiconductor
HQ-Cam	Hight quality Camera (12 megapixel)	2	CAN$53.63	CAN$107.26	Link-6	Semiconductor
FisheyeLens	Arducam M12 mount – 1.56 mm focal length camera lens (M25156H14)	2	CAN$11.99	CAN$23.98	Link-7	Acrylic
LensAdapter	Arducam CS to M12 Adapter for M12 lens and CS lens on Raspberry Pi HQ camera module	2	CAN$3.99	CAN$7.98	Link-8	Nylon
LuxSen	Adafruit VEML7700 lux sensor	6	CAN$5.20	CAN$31.20	Link-9	Semiconductor
IRcam	Adafruit MLX90640 24x32 IR thermal camera breakout – 110-degree FoV	8	CAN$74.95	CAN$599.60	Link-10	Semiconductor
SpecSen	SparkFun triad spectroscopy sensor (AS7265x)	4	CAN$69.95	CAN$279.80	Link-11	Semiconductor
SoundSen	Gravity analog sound level meter with a JST cable	4	CAN$39.50	CAN$158.00	Link-12	Semiconductor
TempHum	Digital temperature and humidity sensor (SHT35)	1	CAN$16.90	CAN$16.00	Link-13	Semiconductor
CO2Sen	MH-Z19B/C infrared CO_2_ Gas Sensor Module with a ribbon cable	1	CAN$60.17	CAN$60.17	Link-14	Semiconductor
PMairSen	PM2.5 Air Quality Sensor and Breadboard Adapter Kit (PMS5003) with a cable	1	CAN$39.95	CAN$39.95	Link-15	Semiconductor
ExpHat	IO Expansion HAT for Raspberry Pi 4B/3B+	1	CAN$9.90	CAN$9.90	Link-16	Semiconductor
Cbl-Cam	Flex Cable for Raspberry Pi Camera or Display (25–30 cm)	2	CAN$1.95	CAN$3.90	Link-17	Conductor
Cbl-i	4-pin female Qwiic cable (15 cm)	16	CAN$1.60	CAN$25.60	Link-18	Conductor
Cbl-ii	4-pin female Qwiic cable (50 cm)	2	CAN$2.10	CAN$4.20	Link-19	Conductor
Cbl-iii	18 AWG Jumper Lead Socket to Socket	1	CAN$2.51	CAN$2.51	Link-20	Conductor
Cbl-iv	Multi-conductor cable	1	CAN$9.63	CAN$9.63	Link-21	Conductor
Cbl-v	4-Pin I2C connector cable	1	CAN$0.95	CAN$0.95	Link-22	Conductor
Hdr-16con	Straight wire wraps male box header, 16 contacts, 2 rows	1	CAN$2.67	CAN$2.67	Link-23	Conductor
Hse-16pos	Connectors housing 16 positions, 2 rows	1	CAN$1.87	CAN$1.87	Link-24	Nylon
Pins-Hse	Female connector pins and housing	1	CAN$11.13	CAN$11.13	Link-25	Metal and nylon
Screw, nut, and spacer						
S-1	Screw – M2, 10 mm length	22	CAN$0.10	CAN$2.20	Link-26	Metal
S-2	Screw – M2, 16 mm length	14	CAN$0.12	CAN$1.68	Link-27	Metal
S-3	Screw – M2.5, 6 mm length	20	CAN$0.15	CAN$3.00	Link-28	Nylon
S-4	Screw – M2.5, 10 mm length	12	CAN$0.11	CAN$1.32	Link-29	Metal
S-5	Screw – M2.5, 10 mm length	8	CAN$0.17	CAN$1.36	Link-30	Nylon
S-6	Screw – M2.5, 12 mm length	4	CAN$0.13	CAN$0.52	Link-31	Metal
S-7	Screw – M3, 6 mm length	16	CAN$0.16	CAN$2.56	Link-32	Nylon
N-1	Nut – M2	2	CAN$0.05	CAN$0.10	Link-33	Metal
N-2	Nut – M2.5	16	CAN$0.07	CAN$1.12	Link-34	Nylon
N-3	Nut – M3	16	CAN$0.06	CAN$0.96	Link-35	Nylon
Sp-1	Spacer – M2, 10 mm length	2	CAN$0.25	CAN$0.50	Link-36	Metal
Sp-2	Spacer – M2.5, 12 mm length	8	CAN$0.30	CAN$2.40	Link-37	Nylon
Sp-3	Spacer – M2.5, 20 mm length	4	CAN$0.35	CAN$1.40	Link-38	Metal
	Total cost			CAN$1,666.96		

## 5 Build instructions

The build instructions are detailed in the following two sub-sections: (5.1) PARSA-360 assembly, and (5.2) PARSA-Air assembly. These instructions are designed to be straightforward and user-friendly, ensuring that students in architecture, design, and building engineering programs can easily reproduce the tool. See (Table 2).

### 5.1 PARSA-360 assembly

**Fig. 2:**
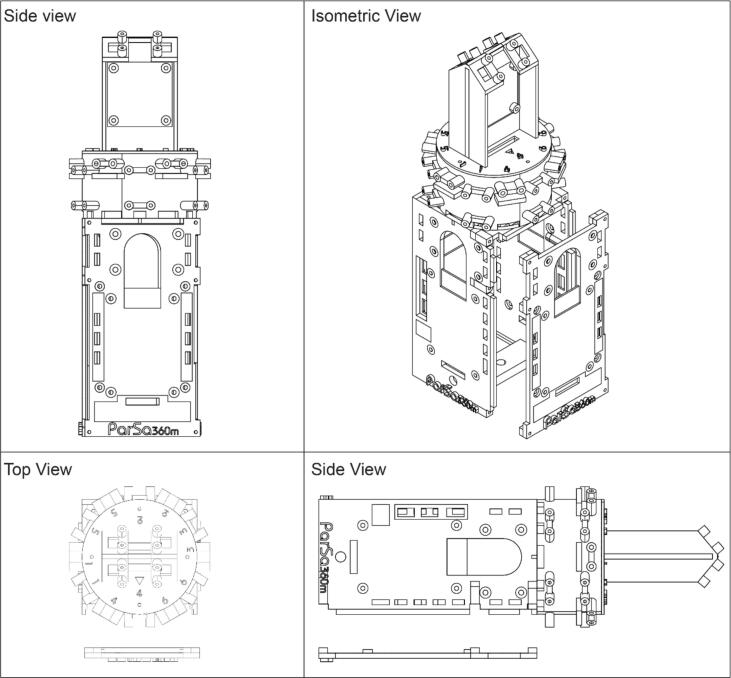
Plastic base box assembly and designated side numbers for PARSA 360 + Air.

The assembly process for PARSA 360 is detailed in the following step-by-step instructions. To ensure consistency and clarity, the four sides of the **PARSA360_BaseBox** square are numbered 1 through 4, as shown in Fig. 2. These numbers are also used to identify the locations of sensors and holes on the circular section of the **PARSA360_BaseBox**. As illustrated in Fig. 2, the diagonal directions and sensors on the circular section are labeled with numbers from 1 to 6. Table 3 presents an overview of the sensors used in the PARSA-360 + Air assembly, highlighting their specific functions and roles within the system.

1. **Mount Sound Level Meters (SoundSen)**
• Attach three SoundSen devices to the PARSA360_BaseBox using M2.5–6 mm nylon screws (S-3) and M2.5 nylon nuts (N-2), as shown in Fig. 3-a.

**Fig. 3:**
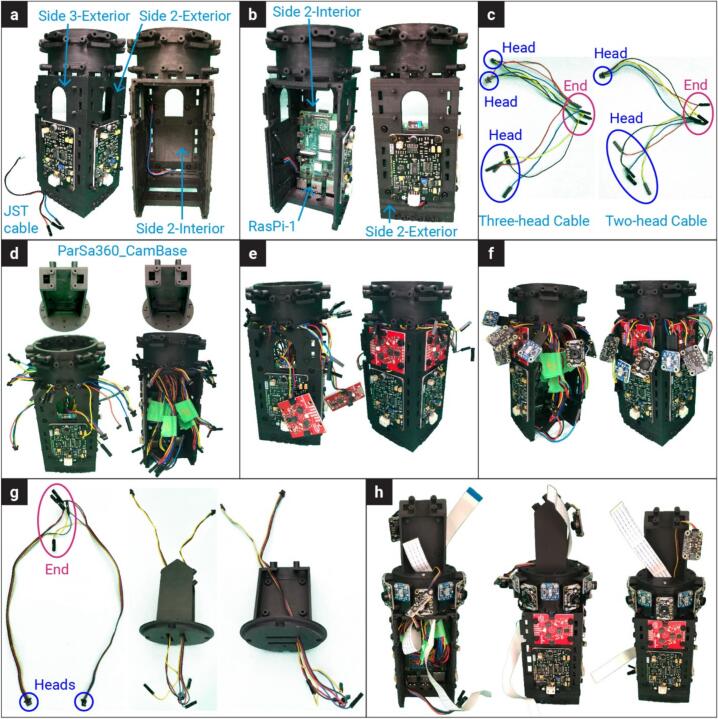
Assembly process of PARSA-360 corresponding to steps 1–30 in in section 5.1.
• Connect the SoundSen cables and route them through the designated holes into the interior.
• Since the original cables are long, it is recommended to cut them to 8–10 cm in length. Re-crimp the cut ends with female connectors.
1. **Install Raspberry Pi 1 (RasPi-1)**
• Mount a Raspberry Pi on the interior surface of the PARSA360_BaseBox using M2.5–10 mm nylon screws (S-5) and spacers (Sp-2), as illustrated in Fig. 3-b.
• For consistency, label this Raspberry Pi as RasPi-1. Ensure it is equipped with a micro-SD card (MicroSD) containing a Debian operating system (OS).
• Position the Raspberry Pi so that the USB and LAN ports face outward, aligning with the bottom of the box.
2. **Create Three-Head Cables**
• Assemble four sets of three-head cables by joining 4-pin female Qwiic cables (Cbl-i), as shown in Fig. 3-c.
• The joined female pins will serve as the cable ends for connection to the expansion hat (ExpHat) in Step 20.
• Prepare the cables as follows:
  o First two sets: Remove the female crimp pins but retain the Qwiic-JST heads for spectrometers (SpecSen) and IR thermal cameras (IRcam).
  o Third set: Remove the Qwiic-JST head but retain the female crimp pins for lux sensors (LuxSen).
  o Join and re-crimp the ends of all three cables with female pins.
• Ensure that each Qwiic cable end includes four wires to connect SDA, SCL, 3.3 V power, and ground pins. Group and re-crimp wires of the same type (e.g., all SDA wires together).
3. **Create Two-Head Cables**
• Assemble two sets of two-head cables by joining 4-pin female Qwiic cables (Cbl-i), as depicted in Fig. 3-c.
• The joined female pins will serve as the cable ends for connection to the ExpHat.
• Prepare the cables as follows:
o First set: Remove the female crimp pins but retain the Qwiic-JST heads for IR thermal cameras (IRcam).
o Second set: Remove the Qwiic-JST head but retain the female crimp pins for lux sensors (LuxSen).
o Join and re-crimp the ends of both cables with female pins, ensuring all SDA, SCL, power, and ground wires are grouped and re-crimped.
4. **Route Three-Head Cables**
• Pass the three-head cables through holes 1–4 on the circular section of the PARSA360_BaseBox (Fig. 3-d).
• Use the large holes for 4-pin female crimp heads and the small holes for Qwiic-JST heads.
• Label each set of cables with a marker according to the designated side numbers (1–4).
5. **Route Two-Head Cables**
• Pass the two-head cables through holes 5 and 6 on the circular section of the PARSA360_BaseBox (Fig. 3-d).
• Use the large holes for 4-pin female crimp heads and the small holes for Qwiic-JST heads.
• Label these cables as 5 and 6 to match the designated side numbers.
6. **Mount SpecSen Devices**
• Attach three SpecSen devices to sides 1, 2, and 3 of the PARSA360_BaseBox using M3-6 mm nylon screws (S-7) and M3 nylon nuts (N-3), as shown in Fig. 3-e.
• For easier installation, place the screw heads inside the box and the nuts on the outside.
7. **Connect Sensors**
• Connect six LuxSen and six IRcams to the Qwiic-JST heads and female crimp pins routed through holes 1–6 (Fig. 3-f).
• Attach SpecSen devices on sides 1–3 to their respective Qwiic-JST heads.
• Note: The Qwiic-JST head from hole 4 remains unconnected for now and will be used for the fourth SpecSen mounted on the PARSA360_Lid in later steps.
8. **Prepare Cables for PARSA360_CamBase**
• Cut two 4-pin female Qwiic cables (Cbl-ii) to 25–30 cm lengths, retaining the Qwiic-JST heads, as shown in Fig. 3-g.
• Crimp female pins to the cut wire ends and route the two-head cables through both sides of the PARSA360_CamBase. These cables are required for IRcams placed on top of the PARSA360_CamBase.
9. **Install PARSA360_CamBase**
• Attach the PARSA360_CamBase to the PARSA360_BaseBox using M2-10 mm metal screws (S-1), as shown in Fig. 3-h.
10. **Mount LuxSen Devices**
• Attach six LuxSen devices to the circular part of the PARSA360_BaseBox using M2.5–10 mm metal screws (S-4), as shown in Fig. 3-h.
• Note: Each LuxSen has two holes for screws.
11. **Mount IRcams**
• Attach six IRcams to the circular part of the PARSA360_BaseBox using M2-10 mm metal screws (S-1).
• Note: Although each IRcam has four screw holes, securing only two diagonal holes is sufficient.
12. **Route Camera Cables**
• Pass two camera cables (Cbl-Cam) through the designated holes on both sides of the PARSA360_CamBase (Fig. 3-h).
13. **Connect and Mount IRcams on PARSA360_CamBase**
• Connect two IRcams to the Qwiic-JST heads on the PARSA360_CamBase.
• Mount the IRcams to their designated positions on top of the PARSA360_CamBase using M2-10 mm metal screws (S-1). Note that screwing only two diagonal holes is sufficient.
14. Mount the Fourth SpecSen and SoundSen on PARSA360_Lid
• Mount the fourth SpecSen and SoundSen on the exterior of the PARSA360_Lid (i.e., side 4 of the PARSA360) as shown in Fig. 4-a.

**Fig. 4:**
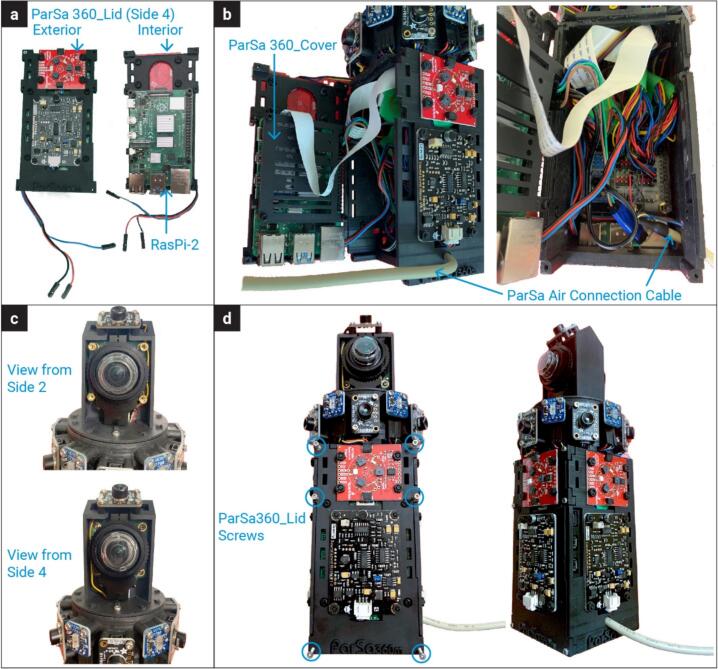
Assembly process of PARSA-360 corresponding to steps 1–30 in section 5.1.
• Ensure that the SoundSen cable is connected properly.
15. Mount Raspberry Pi 2 (RasPi-2) on PARSA360_Lid
• Mount the second Raspberry Pi on the interior of the PARSA360_Lid using M2.5–10 mm nylon screws (S-5) and spacers (Sp-2), as illustrated in Fig. 4-a.
• Label this Raspberry Pi as RasPi-2.
• Insert a MicroSD card with the Debian OS into RasPi-2.
• Pass the SoundSen cable through the available space between RasPi-2 and the lid.
• Position the Raspberry Pi so that the USB and LAN ports face outward, aligned with the bottom of the lid.
16. Connect Camera Cables to RasPi-1 and RasPi-2
• Connect the Camera-1 cable to RasPi-1 and the Camera-2 cable to RasPi-2 (Fig. 4-b).
• Once the Camera-2 cable is connected to RasPi-2, secure the PARSA360_Cover onto RasPi-2.
17. Mount the Expansion Hat (ExpHat) on RasPi-1
• Mount the ExpHat on RasPi-1.
• Pass the Camera-1 cable through the available space between the ExpHat and RasPi-1 (Fig. 4-b).
18. Connect Qwiic Cables to the ExpHat
• Connect all three-head and two-head Qwiic cables to the ExpHat according to the pin and I2C bus layout shown in Fig. 5 and Table 4.

**Fig. 5:**
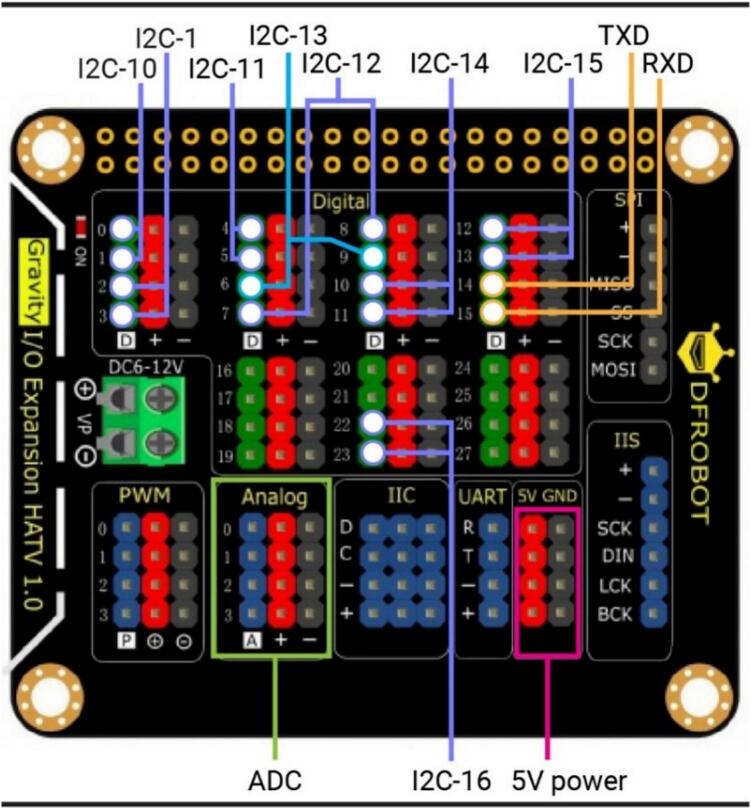
Expansion Hat pin layout showing the I2C buses designated for sensors. The numbers next to the pins in the digital section correspond to the GPIO numbers.

**Table 4:** I2C bus and GPIO pin settings on the ExpHat for connecting three-head and two-head Qwiic cables for LuxSens, IRcams, and SpecSens.

**Cable No.**	**Bus No.**	**SDA (GPIO No.)**	**SCL (GPIO No.)**
IRcam-1	1	2	3
IRcam-2	10	0	1
1 (Three-head)	11	4	5
2 (Three-head)	12	8	7
3 (Three-head)	13	6	9
4 (Three-head)	14	10	11
5 (Two-head)	15	12	13
6 (Two-head)	16	22	23
• Each Qwiic cable end contains SDA, SCL, 3.3 V power, and ground wires.
• Connect the SDA and SCL wires of the three-head Qwiic cables (sides 1–4) to the I2C bus pins 11–14, respectively.
• Connect the SDA and SCL wires of the two-head Qwiic cables (sides 5 and 6) to the I2C bus pins 15 and 16, respectively.
• Connect the power and ground wires to any available 3.3 V power and ground pins on the digital section of the ExpHat.
19. Connect Qwiic Cables for IRcams on PARSA360_CamBase
• Connect the Qwiic cables from the IRcams mounted on top of the PARSA360_CamBase to I2C bus pins 1 and 10 on the ExpHat.
• Power and ground wires can be connected to any available 3.3 V power and ground pins on the digital section of the ExpHat.
20. Connect SoundSen Cables to ADC Pins on the ExpHat
• Connect the SoundSen cables to the analog-to-digital converter (ADC) pins on the ExpHat, as shown in Fig. 5.
• The SoundSen devices require an ADC to function, which is provided by the ExpHat with four available ADC channels.
• Connect the SoundSen devices on sides 1–4 to ADC channels 0–3, respectively.
• Connect the power and ground wires for each SoundSen to the designated pins on the ADC section of the ExpHat.
21. Prepare Multi-Conductor Cable (Cbl-iv)
• Cut the multi-conductor cable (Cbl-iv) to a length of approximately 20 cm.
• Crimp a female pin to one end of the cable and pass it through the circular hole designated on either side 1 or side 3 of the PARSA360_BaseBox, as shown in Fig. 4-b.
• The multi-conductor cable connects the PARSA Air unit to the PARSA360 and includes nine wires: SDA, SCL, 3.3 V power, 5 V power, two Ground wires, and two RXD and TXD wires.
22. Connect 5 V Power and Ground of Cbl-iv to ExpHat
• Connect the 5 V power and ground wires of the Cbl-iv to the appropriate 5 V power and ground pins on the ExpHat (Fig. 5).
23. Connect 3.3 V Power and Ground of Cbl-iv to ExpHat
• Connect the 3.3 V power and ground wires of the Cbl-iv to the corresponding 3.3 V power and ground pins on the ExpHat (Fig. 5).
24. Connect RXD and TXD Wires of Cbl-iv to ExpHat
• Connect the RXD and TXD wires of the Cbl-iv to their respective RXD and TXD pins on the ExpHat, as shown in Fig. 5.
• These wires will be used to interface with the CO_2_ sensor (CO2Sen).
25. Connect SDA and SCL Wires of Cbl-iv to RasPi-2
• Connect the SDA and SCL wires of the Cbl-iv to the respective SDA and SCL pins on RasPi-2 (Fig. 6).

**Fig. 6:**
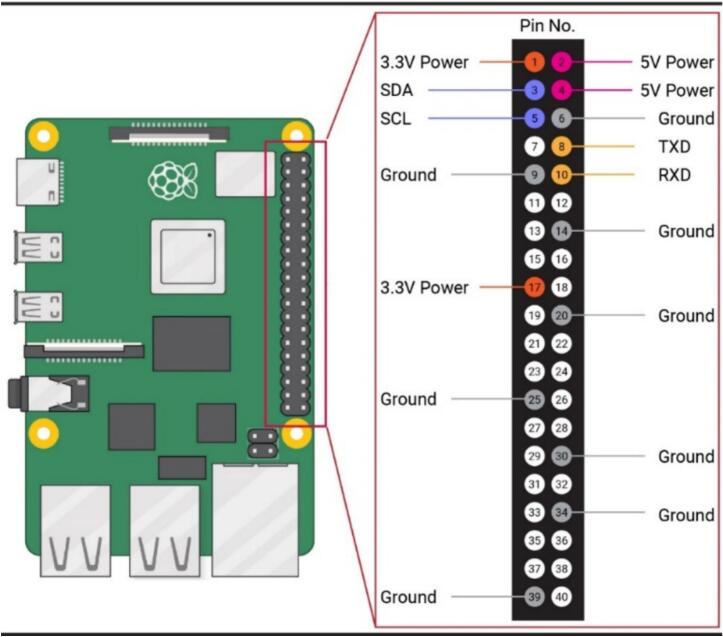
Raspberry Pi pinout, with the numbers in circles representing the actual pin numbers.
26. Connect RXD Wire of Cbl-iv to RasPi-2
• Connect the RXD wire of the Cbl-iv to the RXD pin on RasPi-2 (Fig. 6).
• This wire will be used to interface with the PM meter (PMairSen).
27. Use Jumper Cable to Connect Power and Ground between RasPi-2 and ExpHat
• Use an 18 AWG jumper cable (Cbl-iii) to connect the 5 V power and ground pins on RasPi-2 to the corresponding 5 V power and ground pins on the ExpHat (Figs. 5 and 6).
• You may need to cut and re-crimp the Cbl-iii to a length of approximately 10–15 cm with female pins. This cable ensures both Raspberry Pis receive power from a single external supply.
28. Mount and Secure HQ-Cams
• Mount the high-quality cameras (HQ-Cam) using M2.5–12 mm metal screws (S-6) and M2.5–20 mm metal spacers (Sp-3), as shown in Fig. 4-c.
• Ensure both cameras are mounted simultaneously, with screws passing through one camera board to reach spacers on the other camera board.
• Install fisheye lenses on the cameras.
29. Close and Secure PARSA360_Lid
• Close the PARSA360_Lid and secure it using M2-16 mm metal screws (S-2), as illustrated in Fig. 4-d.

**Table 3:** Overview of sensors in PARSA-360 + Air and their functions.

**Sensor Name**	**Function**
Hight quality Camera (12 megapixel) mounted with Arducam M12 mount – 1.56 mm focal length lens (M25156H14)	Captures panoramic photos to generate High Dynamic Range (HDR) images and analyze pixel-based photobiological lighting characteristics and spatial attributes
Adafruit MLX90640 24x32 IR thermal camera breakout – 110-degree FoV	Captures infrared thermographic images to assess thermal characteristics
SparkFun triad spectroscopy sensor (AS7265x)	Measures spectral power distribution of light for photobiological lighting analysis
Adafruit VEML7700 lux sensor	Measures light illuminance intensity for photobiological lighting analysis
Digital temperature and humidity sensor (SHT35)	Monitors ambient air temperature and humidity to evaluate thermal conditions
MH-Z19B/C infrared CO_2_ Gas Sensor	Measures CO_2_ concentration to assess air quality
PM2.5 Air Quality Sensor and Breadboard Adapter Kit (PMS5003)	Measures the concentration of air particulate matter (PM) to evaluate air quality
Gravity analog sound level meter	Measures sound intensity for acoustic analysis

### 5.2 PARSA-Air assembly

The PARSA-Air unit assembly is explained in the following step-by-step instructions.

2. Install the straight wire wraps male box header (Hdr-16con) at the designated location on the PARSAAir_BaseBox using M2-10 mm metal screws (S-1), as illustrated in Fig. 7-a.

**Fig. 7:**
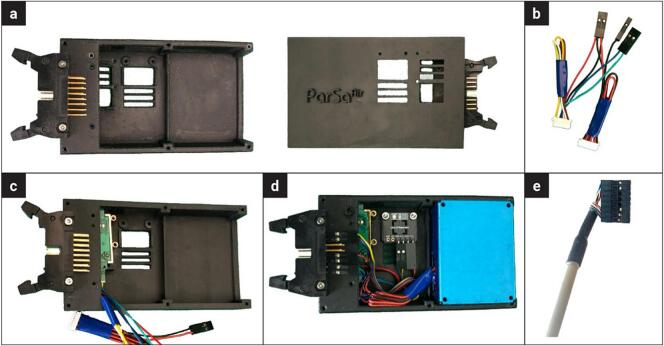
Assembly process of PARSA-Air corresponding to steps 1–10 in section 5.2.
3. Join and re-crimp the power and ground wires of the CO2Sen and PMairSen sensors, as shown in Fig. 7-b. Crimp the TXD and RXD wires of the CO2Sen with female pins as they are required. The remaining wires of the CO2Sen can be cut or concealed. Crimp the TXD wire of the PMairSen with a female pin and cut or conceal the other wires.
4. Place the CO2Sen at the designated location in the PARSAAir_BaseBox, as shown in Fig. 7-c. Note that the CO2Sen cable should already be connected.
5. Mount the TempHum sensor at its designated location using M2-10 mm metal screws (S-1) and M2 metal nuts (N-1), as illustrated in Fig. 7-d.
6. Connect the TempHum sensor pins (i.e., SDA, SCL, 3.3 V power, and ground) to the Hdr-16con connector using a short 4-wire cable (approximately 5 cm) with female heads on both ends, as shown in Fig. 7-d.
7. Connect the PMairSen cable and place it at the designated location in the PARSAAir_BaseBox.
8. Connect the CO2Sen-PMairSen joined cable to the Hdr-16con connector, as shown in Fig. 7-d.
9. Crimp female pins to the Cbl-iv wires and add the 16-position connector housing (Hse-16pos), as shown in Fig. 7-e. Carefully place the Cbl-iv wires, as detailed in Step 22 of the previous section, so that they match with the corresponding Hdr-16con pins. This means that the 3.3 V power, 5 V power, ground, SDA, and SCL wires of the Cbl-iv should be connected to the same pins and respective wires on the Hdr-16con. However, the RXD and TXD wires should be cross-connected: the RXD wire from the Cbl-iv should be connected to the TXD pin, and the TXD wire from the Cbl-iv should be connected to the RXD pin.
10. Mount and screw the PARSAAir_Lid using M2-16 mm metal screws (S-2).
11. Connect the air unit to the PARSA-360 unit via the Cbl-iv, as shown in Fig. 1.

## 6 Operation instructions

**Fig. 8:**
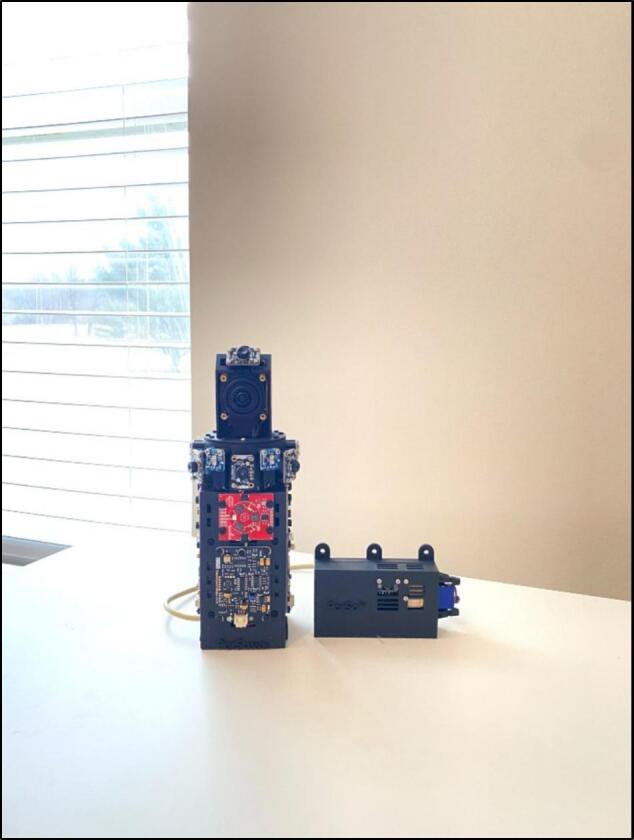
PARSA-360 + Air set up as a stationary system on a desk.

PARSA 360 + Air (both units) powers on by connecting a power supply to any of the Raspberry Pis (RasPis). As highlighted in the previous assembly instructions, both RasPis should already have MicroSD cards loaded with the Debian OS. PARSA-360 + Air can be used as a stationary system placed on a desk (Fig. 8) or mounted on a tripod.

For first-time setup, the required libraries, dependencies, and programs must be downloaded and installed. All necessary libraries and files are available for download and installation on the PARSA 360 + Air GitHub page [46] (see Table 1). Installing the required libraries is straightforward by following the instructions on the GitHub page. The command lines provided in the GitHub instructions should be executed on each RasPi-1 and RasPi-2 to download the necessary libraries. Next, the ‘Installation.sh’ script should be executed to install all dependencies and perform system updates on the Raspberry Pi devices used in PARSA-360 + Air. This process includes the following tasks:

• Install dependencies, including Pandas, thermal cameras, imagery cameras, CO_2_, and PM sensors.
• Configure I2C ports and update and upgrade OS packages.
• Move the file ‘PARSA360Air_LoggerRun.py’ to the home directory.

**Fig. 9:**
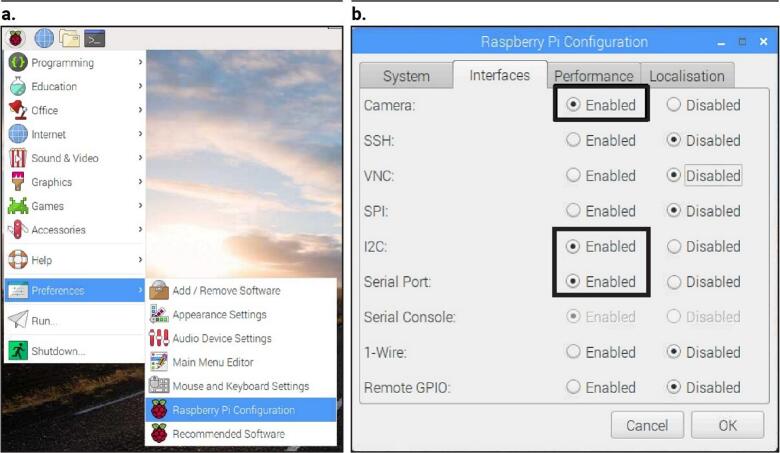
Activation of Raspberry Pi's camera module, I2C, and serial ports.

The camera modules, I2C, and serial connections are activated on both RasPis, as shown in Fig. 9. Additional instructions can be found in the Raspberry Pi Foundation [48], Adafruit [49], and Hawkins [50] resources. After installation and activation are complete, reboot the RasPis.

Once the RasPis are ready, run PARSADataLoggerSet.py on each RasPi to start capturing imagery and multisensory data. The script will prompt for five specific inputs as explained in the following. The running processes have been simplified to make them easier for potential users.

1. **Set Raspberry Pi**
• The input can either be 1 or 2, based on the following criteria:
o If the script is running on RasPi-1, input 1.
o If the script is running on RasPi-2, input 2.
2. **Set the entire duration of measurement (in minutes)**
• This input specifies the total duration for the imagery and sensory measurement process. Fig. 10 provides a graphical example for clarification.

**Fig. 10:**
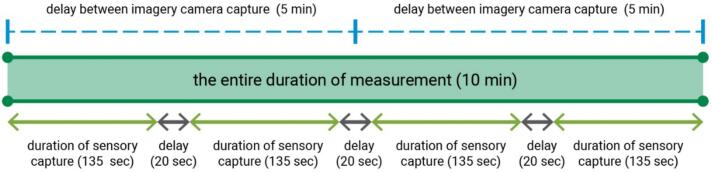
Example of time inputs for imagery and multisensor data capture by PARSA-360 + Air.
3. **Set the duration of each sensory capture (in seconds)**
• This input defines the duration for each sensory module (including IRcam, LuxSen, SpecSen, SoundSen, TempHum, CO2Sen, and PMairSen) to measure related data. Once the set duration ends, the average measurements from each module are automatically saved in the ‘DataLogger’ directory on each RasPi (Fig. 10).
4. **Set the delay between each sensory capture (in seconds)**
• This input defines the sleep time between each sensory module capture (Fig. 10).
5. **Set the delay between each imagery camera capture (in minutes)**
• This input defines the sleep time between each imagery camera capture (Fig. 10). The camera captures will automatically be saved in the ‘DataLogger’ directory on each RasPi.

**Fig. 11:**
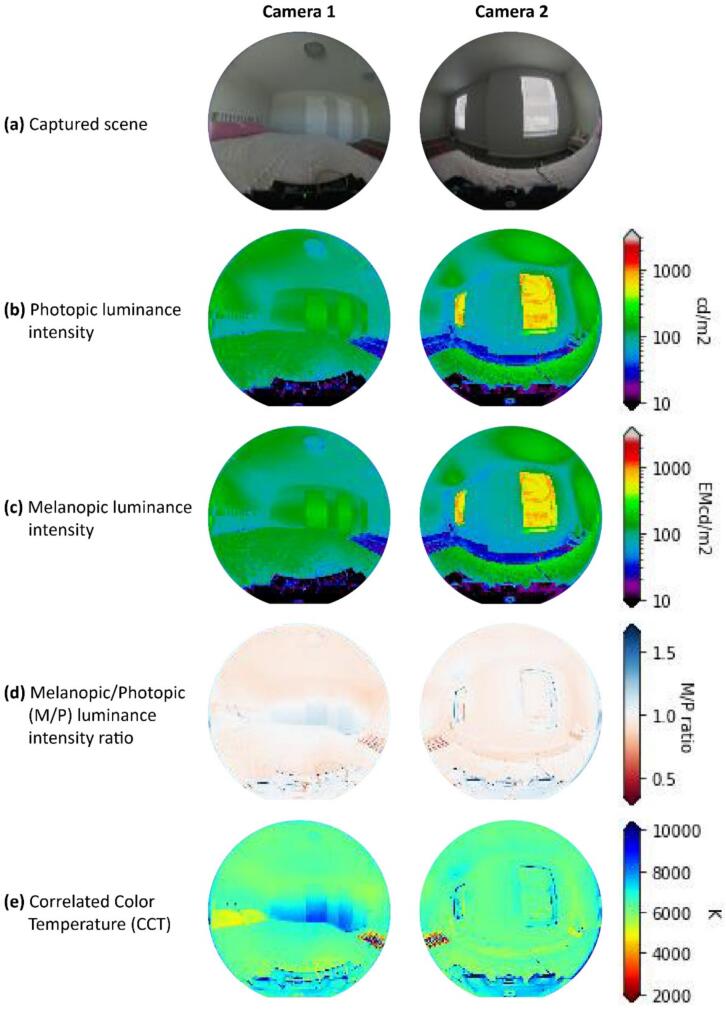
An example of tone-mapped HDR images captured by PARSA-360 + Air cameras along with corresponding photobiological analysis: (a) camera-captured scenes, (b) false color maps of photopic luminance intensity of the captured scenes, (c) false color maps of melanopic luminance intensity of the captured scenes, (d) melanopic/photopic (M/P) luminance intensity ratio of the captured scenes, (e) false color maps of correlated color temperature (CCT) for the captured scenes.

**Fig. 12:**
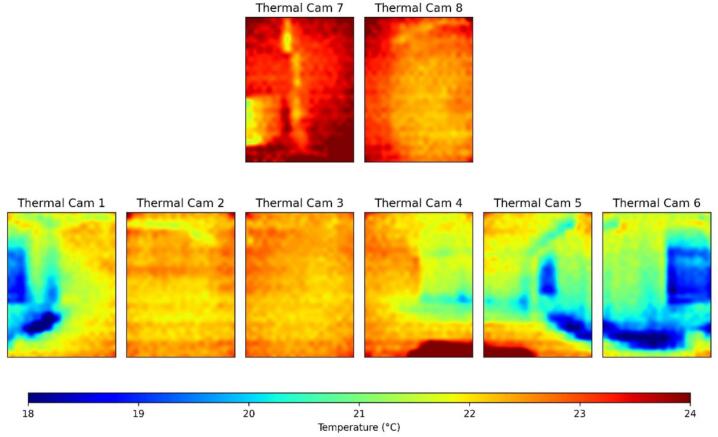
An example of thermographic images captured by PARSA-360 + Air infrared thermographic sensors.

**Fig. 13:**
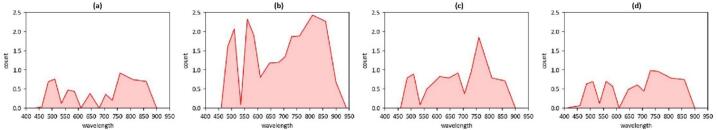
An example of spectral power distribution data captured by the PARA 360 + Air spectroscopy sensors from four directions, creating a panoramic view. The views are: (a) 0–90 degrees, (b) 90–180 degrees, (c) 180–270 degrees, and (d) 270–360 degrees.

**Fig. 14:**
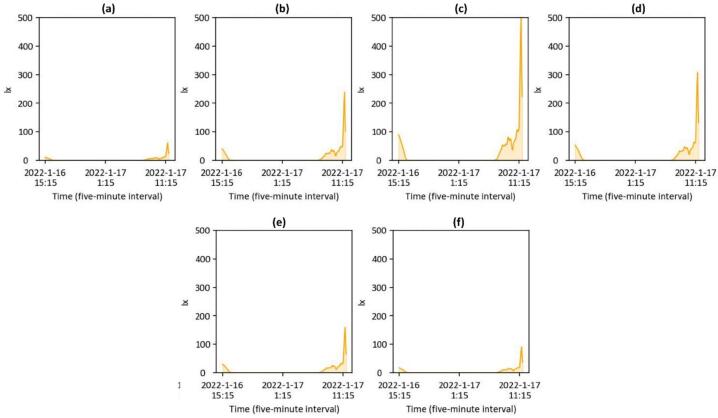
An example of lighting illuminance levels captured by the PARA 360 + Air illuminance meter sensors from four directions, creating a panoramic view. The views are: (a) 0–60 degrees, (b) 60–120 degrees, (c) 120–180 degrees, (d) 180–240 degrees, (e) 240–300 degrees, and (f) 300–360 degrees.

**Fig. 15:**
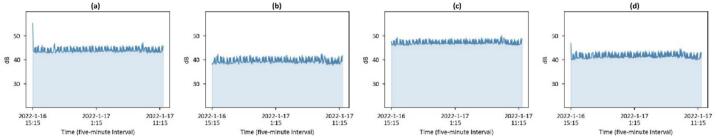
An example of sound levels captured by the PARA 360 + Air sound level meter sensors from four directions, creating a panoramic view. The views are: (a) 0–90 degrees, (b) 90–180 degrees, (c) 180–270 degrees, and (d) 270–360 degrees.

**Fig. 16:**
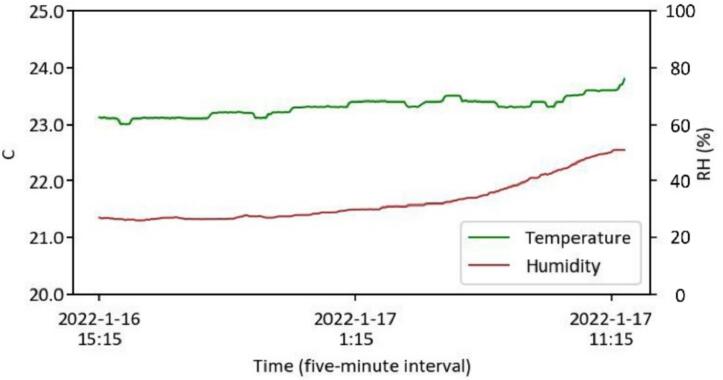
An example of ambient air temperature and humidity levels captured by the PARA 360 + Air hygro-thermometer sensor.

**Fig. 17:**
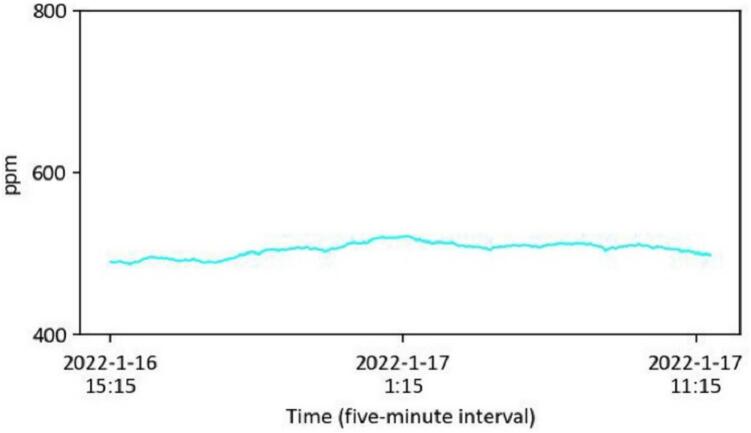
An example of ambient air carbon dioxide (CO_2_) concentration captured by the PARA 360 + Air CO_2_-meter sensor.

**Fig. 18:**
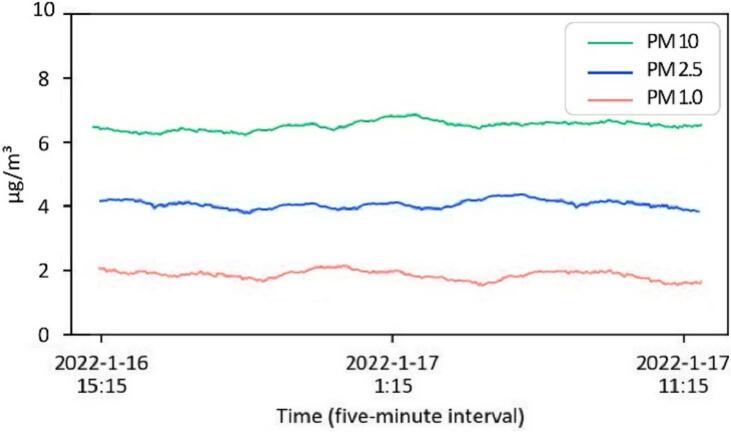
An example of ambient air particulate matter (PM) concentrations captured by the PARA 360 + Air PM sensor, measuring PM1 (particles with a diameter of 1 µ m or less), PM2.5 (particles with a diameter of 2.5 µ m or less), and PM10 (particles with a diameter of 10 µ m or less).

The data capturing and storage process is customizable through the Python code provided in the ‘libs’ folder. A basic knowledge of Python programming is required. The captured data is stored locally on each Raspberry Pi. Users can design and develop their own code to modify how they collect and share data. Users can also visualize and process the data based on environmental needs for comfort assessment. As detailed in Table 1, a Python library has been developed and shared on GitHub [47], enabling basic visualization of the captured data. Users can clone the files via the link provided in Table 1, with instructions available on the page. Briefly, Fig. 11, Fig. 12, Fig. 13, Fig. 14, Fig. 15, Fig. 16, Fig. 17, Fig. 18 visualize some of the data captured by PARSA 360 + Air and plotted using the developed Python library.

## 7 Validation and characterization

PARSA-360 + Air is built using low-cost sensors and camera technologies, offering sufficient accuracy for environmental analysis in architectural and urban spaces. The modules deliver satisfactory performance for educational purposes in architectural, urban design, and building engineering programs. Additionally, they can be further calibrated by specialized researchers if needed. Table 5 presents the technical specifications and operational range of the modules used in PARSA-360 + Air.

**Table 5:** Technical specifications and operational range of the modules used in PARSA-360 + Air.

**Sensor**	**Manufacturer/ Supplier**	**Specification**
AS7265x Spectroscopy Sensor	SparkFun [53]	• 18 frequencies of light sensing from 410 nm to 940 nm • 28.6 μW/cm^2^ per count with a channel count accuracy of ± 12 % per μW/cm^2^ • −40 °C to + 85 °C operating temperature range • 2.7 V to 3.6 V operating voltage range with I^2^C interface
VEML7700 Ambient Light	Adafruit [52] and Vishay [51]	• 16-bit dynamic range for ambient light detection from 0 lx to about 120 klx with resolution down to 0.0036 lx/ct, • supports low transmittance (dark) lens design • 100 Hz and 120 Hz flicker noise rejection • Excellent temperature compensation • High dynamic detection resolution • −25 °C to + 85 °C operating temperature range • 3.3 V to 5 V operating voltage range with I^2^C interface
DFRobot Gravity Analog Sound Level Meter	DFRobot [55]	• Measuring Range: 30dBA ∼ 130dBA • Measurement Error: ±1.5 dB • Frequency Response: 31.5 Hz ∼ 8.5KHz • Time Characteristics: 125 ms • Input Voltage: 3.3 ∼ 5.0 V • Output Voltage: 0.6 ∼ 2.6 V
MLX90640 24x32 IR Thermal Camera Breakout − 110-Degree Field of view	Adafruit [52]	• I2C compatible digital interface • Programmable refresh rate 0.5 Hz…64 Hz (0.25 ∼ 32 FPS) • 3.3 V-5 V supply voltage • Field of view: 110°x70° • Operating temperature −40 °C to + 85 °C • Target temperature −40 °C to + 300 °C
PM2.5 Air Quality Sensor	Adafruit [52]	• Range of measurement: 0.3 ∼ 1.0；1.0 ∼ 2.5；2.5 ∼ 10 μ m • Effective Range: 0 ∼ 500 μ g/m^3^ • Maximum Range: * ≥1000 μ g/m^3^ • Resolution: 1 μ g/m^3^ • Power Supply: Typ:5.0 / Min:4.5 / Max: 5.5 V • Working Temperature Range: −10∼+60 ℃ • Working Humidity Range: 0 ∼ 99 % • Storage Temperature Range: −40∼+80 ℃
MH-Z19C carbon dioxide gas sensor	Winsen [56]	• Supply voltage: 4.5 ∼ 5.5 V • Measurement range: 400 ∼ 5000 ppm • Detection accuracy: ± 50 ppm to ± 5 % reading value • Warm-up time: 1 min • Working temperature: 0–––50 °C • Operating Humidity: 0 to 95 % RH (non-condensing) • Storage temperature: −20 ∼ 60 °C
DFRobot Gravity Digital Temperature & Humidity Sensor (SHT31-F)		• Operating Voltage: 3.3 ∼ 5.5 V • Humidity Detection Range: 0 %RH ∼ 100 %RH • Humidity Accuracy: ±2%RH at 10 %RH ∼ 90 %RH (at 25 °C) • Temperature Detection Range: −40 ∼ 125 °C • Temperature Accuracy: ±0.2 °C at 0 ∼ 90 °C (Typical) • Communication: I2C
Raspberry Pi High Quality (HQ) Camera	Raspberry Pi [57]	• Sensor: Sony IMX477 • 12.3 megapixels • Sensor resolution: 4056 x 3040 pixels • 6.287 mm x 4.712 mm (7.9 mm diagonal) • 1.55 μm × 1.55 μm pixel size • Output: RAW12/10/8, COMP8 • Back focus: Adjustable (12.5 mm–22.4 mm) • Lens standards: C-mount • CS-mount (C-CS adapter included) • IR cut filter: Integrated • Ribbon cable length: 200 mm • Tripod mount: 1/4″-20
Arducam 180 Degree Fisheye M12	Arducam [58]	• Optical Format: 1/2.3″ • Focal Length: 1.56 mm • Aperture: F2 • Field of View (FOV): 180° (H) on Raspberry Pi High Quality Camera, 140°(H) on Raspberry Pi V1/V2 Camera • Mount: M12 mount • Back Focal Length: 4.3 mm • MOD: 0.2 *m* • IR Sensitivity: sensitive to visible light, with 650 nm IR filter

**Fig. 19:**
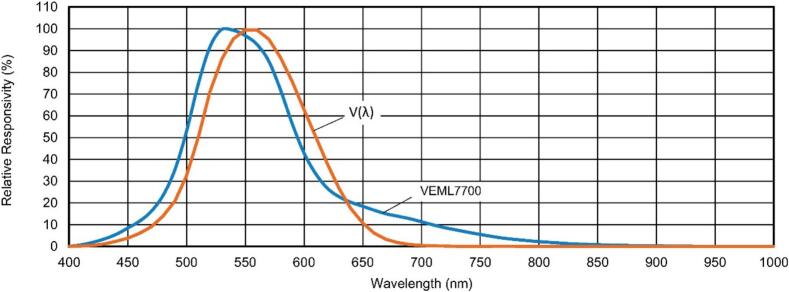
Spectral sensitivity of the VEML7700 compared to the photopic (V(λ)) curve [51], [52].

**Fig. 20:**
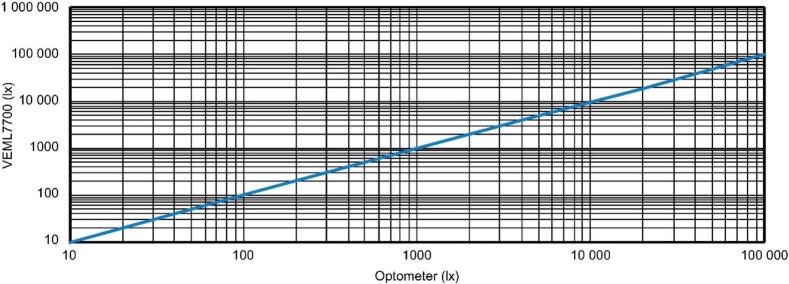
Linear response of the VEML7700 across a range of lux levels from 0.0042 lx to 100 klx [51], [52].

The VEML7700 spectral sensitivity closely follows the photopic (v(λ)) curve as shown in Fig. 19 [51], [52]. The VEML7700 also exhibits good linear behavior for lux levels ranging from 0.0042 lx to approximately 100 klx, as illustrated in Fig. 20 [51], [52].

**Fig. 21:**
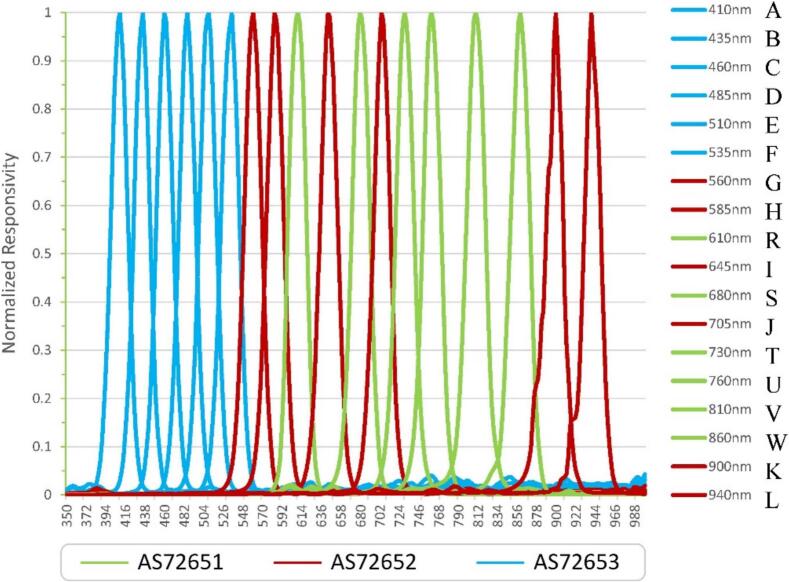
The AS7265x 18-channel spectral sensor system consists of three devices (AS72651, AS72652, and AS72653) covering wavelengths from 410  nm to 940  nm with a Full Width at Half Maximum (FWHM) of 20  nm. The AS72651 covers 600  nm to 870  nm, AS72652 spans 560  nm to 940  nm, and AS72653 ranges from 410  nm to 535  nm [53].

The AS7265x spectroscopy sensor chipset consists of three sensor devices: AS72651 (with master capability), AS72652, and AS72653 [53]. As shown in Fig. 21, the AS7265x multispectral sensor enables spectral identification across a range from visible to near-infrared (NIR). Each of the three sensor devices is equipped with six independent optical filters. These filters cover the spectral range from 410  nm to 940  nm, each with a Full Width at Half Maximum (FWHM) of 20  nm. The AS72651 serves as the master controller and covers wavelengths from 600  nm to 870  nm. When combined with the AS72652 (covering 560  nm to 940  nm) and AS72653 (covering 410  nm to 535  nm), the full set delivers 18 channels, enabling detailed multispectral analysis across visible to near-infrared wavelengths (Fig. 21). These components are pre-calibrated with a specific light source, ensuring a channel count accuracy of ± 12 % per μW/cm^2^ [53]. Calibration conditions, including light source, gain, and integration time, are detailed in the optical characteristics table for each component [53]. Further studies on sensitivity, accuracy, and calibration methods can be found in Amirazar, et al. [8], Botero-Valencia, et al. [9].

The DFRobot Gravity Analog Sound Level Meter is a factory-calibrated module with a measuring range of 30 dBA to 130 dBA and a frequency response of 31.5 Hz to 8.5 kHz. However, this sensor does not cover the full human hearing range of approximately 20 Hz to 20 kHz. Future users may consider replacing it with a sensor that offers a wider range or combining multiple sensors to achieve broader frequency coverage.

**Fig. 22:**
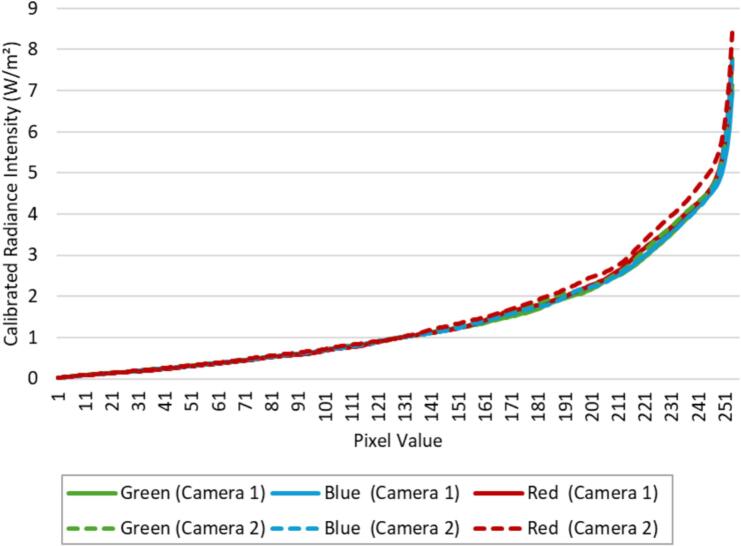
Response curves generated for cameras 1 and 2. Note that pixel values typically range from 0 to 255 for 8-bit images, representing the intensity levels of each color channel. These values are unitless integers that correspond to the brightness levels captured by the camera sensor.

The Python scripts developed for PARSA-360 + Air generate and calibrate HDR images based on the camera response function (CRF) formulated by Debevec and Malik [54] (Fig. 22). The CRF establishes the relationship between scene radiance and measured intensity values. The CRF is represented by a 256-length vector for each color channel, i.e., red, blue, and green. Luminance maps of the captured HDR scenes can be generated and validated more accurately using the methods described by Pierson, et al. [31]. If needed, further photometric calibration of HDR images is explained in [27], [28], [30], [31]. Note that the CRF and other HDR photometric parameters are specific to the camera and lens.

### 7.1 Conclusion

The PARSA-360 + Air system introduces an innovative, low-cost, and customizable approach for capturing and visualizing environmental parameters within architectural and urban spaces. By integrating 360-degree imagery with multi-sensor data collection, this system offers a comprehensive and immersive method for assessing lighting, thermal conditions, air quality, acoustics, and spatial attributes. Unlike existing tools that focus on isolated parameters, PARSA-360 + Air enables simultaneous environmental data acquisition, facilitating a holistic understanding of built environments. The open-source nature of the system, combined with its modular design and straightforward assembly process, makes it accessible to researchers, educators, students, and practitioners in architecture, building engineering, and urban design. Future research and development could focus on expanding the system's IoT integration, enhancing sensor calibration for improved accuracy, and exploring long-term environmental monitoring applications. Additionally, studies could investigate the system’s capacity to support deep-learning workflows and augmented/mixed reality applications, offering new opportunities to advance environmental analysis, human-centered design, and interactive learning experiences. Overall, the adaptability of PARSA-360 + Air paves the way for continued innovation in environmental sensing, providing valuable insights for creating healthier, more sustainable, and user-responsive built environments.

## 8 Ethics statements

The presented datasets do not involve any human subjects, or animal experiments, or using social media platforms.

## CRediT authorship contribution statement

**Mojtaba Parsaee:** Writing – review & editing, Writing – original draft, Visualization, Validation, Software, Methodology, Investigation, Formal analysis, Data curation, Conceptualization. **André Potvin:** Writing – review & editing, Supervision, Methodology, Funding acquisition. **Jean-François Lalonde:** Writing – review & editing, Supervision, Funding acquisition. **Marc Hébert:** Writing – review & editing, Supervision, Funding acquisition. **Claude M.H. Demers:** Writing – review & editing, Supervision, Project administration, Funding acquisition, Conceptualization.

## Declaration of competing interest

The authors declare that they have no known competing financial interests or personal relationships that could have appeared to influence the work reported in this paper.
